# Septic Shock and RAAS Dysregulation: Corticosteroids Don’t Tell the Whole Story

**DOI:** 10.1016/j.aicoj.2025.100001

**Published:** 2025-10-25

**Authors:** Bruno Garcia, Adrien Picod, Camille Benaroua, Fabio Silvio Taccone, Filippo Annoni

**Affiliations:** a Department of Intensive Care, Erasme Hospital, Hôpital Universitaire de Bruxelles, Université Libre de Bruxelles (ULB), Brussels, Belgium; b Department of Intensive Care, Centre Hospitalier Universitaire de Lille, Lille, France; c French Clinical Research Infrastructure Network - Cardiovascular and Renal Clinical Trialists (F-CRIN INI-CRCT), France; d Medical-Surgical Intensive Care Unit, Hôpital Avicenne, AP-HP, Bobigny, France; e Université Paris Cité, UMR-S 942, INSERM, MASCOT, Paris, France

Dear Editor,

We thank Uslar et al. for their thoughtful comments on our study addressing alterations renin–angiotensin–aldosterone system (RAAS) in septic shock [1]. While we appreciate the hypotheses proposed by the authors, we wish to underscore several pathophysiological considerations which, in our opinion, do not fully account for the RAAS alterations observed in our cohort.

The authors proposed that hydrocortisone, at pharmacological doses, may activate mineralocorticoid receptors (MR) and suppress renin and aldosterone release through negative feedback, an effect potentially amplified by concomitant fludrocortisone. However, numerous studies have demonstrated that renin levels are markedly elevated in septic shock, while aldosterone concentrations remain often disproportionately low relative to renin. If corticosteroids substantially modulate this axis, a decrease in renin levels would be expected, yet this is supported by recent evidence, showing persistently high renin concentrations in septic shock [2]. Furthermore, during septic shock, a relatively low dose of 200 mg of hydrocortisone is administered for few days, whereas aldosterone release was reported to be significantly reduced only after ten weeks of treatment at high dose (0.4–0.6 mg/Kg) compared to lower doses (0.2–0.3 mg/Kg) [3].

The letter also suggests that glucocorticoids may upregulate hepatic angiotensinogen synthesis, potentially increasing angiotensin I independently of renin concentration. While this mechanism is theoretically plausible and neither angiotensinogen concentration nor renin concentration were measured in our cohort, it is not consistent with recent data. A multicenter cohort study of sepsis and septic shock reported that circulating intact angiotensinogen concentration is decreased and more strongly associated with 30-day mortality than renin or lactate, indicating that low angiotensinogen reflects increased consumption driven by high renin. These findings further support the concept that the rise in angiotensin I during septic shock is primarily mediated by increased renin secretion rather than by enhanced angiotensinogen production [4].

Uslar et al. propose that glucocorticoids may upregulate angiotensin-converting enzyme 2 (ACE2), thereby shifting the RAAS toward the alternative ACE2/Angiotensin-(1–7) axis. However, the shift toward the alternative RAAS has been reported both in experimental and clinical studies, independently of corticosteroid exposure. In a large swine model of septic shock resuscitated with fluids, vasopressors, and antibiotics but without corticosteroids, we observed increased Angiotensin I/II and Angiotensin-(1–7)/Angiotensin II ratios, together with elevated Angiotensin-(1–7) concentrations, all related to septic shock [5]. Likewise, a *post hoc* analysis of the VICTAS trial reported increased ACE2 activity irrespective of corticosteroid use, suggesting that this shift represents a sepsis-related mechanism rather than a corticosteroid-driven effect [6].

The letter interprets the changes observed at 6 h as a rapid corticosteroid effect on RAAS disturbances. However, RAAS abnormalities were already present at baseline (H0). Moreover, several studies have demonstrated substantial variability in plasma fludrocortisone concentrations, likely reflecting inconsistent intestinal absorption and variable hepatic metabolism [7]. It is therefore unlikely that the early RAAS abnormalities observed in our cohort were meaningfully influenced by hydrocortisone or fludrocortisone administration within such a short timeframe. Importantly, corticosteroids remain among few interventions associated with improved survival in septic shock and are recommended by international guidelines. This makes it particularly challenging to analyze the RAAS profile in septic shock independently from corticosteroids use, and perhaps of limited clinical utility.

Lastly, it is plausible that the RAAS modifications during sepsis would not equal the ones during septic shock, and it is possible that the impact of the exogenous administration of corticoids may differ according to the time of administration.

## CRediT authorship contribution statement

BG drafted and wrote the manuscript. All authors critically reviewed the content and approved the final version for submission.

## Consent for publication

Not applicable.

## Ethics approval and consent to participate

Not applicable. This article is a commentary on previously published work and did not involve any new studies with human participants or animals.

## Funding

No external funding was received for this work.

## Availability of data and material

Not applicable.

## Declaration of competing interest

The author declares no competing interests related to this work.
